# Polymer Interface Molecular Engineering for E-Textiles

**DOI:** 10.3390/polym10060573

**Published:** 2018-05-23

**Authors:** Chuang Zhu, Yi Li, Xuqing Liu

**Affiliations:** School of Materials, University of Manchester, Oxford Road, Manchester M13 9PL, UK; chuang.zhu@postgrad.manchester.ac.uk (C.Z.); henry.yili@manchester.ac.uk (Y.L.)

**Keywords:** electroless deposition, polymer interface molecular engineering, wearable electronics, conductive fibres

## Abstract

Wearable electronics, regarded as the next generation of conventional textiles, have been an important concept in the study of e-textiles. Conductive fibres are the upstreaming of e-textiles and have witnessed the booming development in recent years. However, little work has focused on improving the wash ability and durability of conductive fibres. As a new approach to manufacturing conductive fibres, Polymer Interface Molecular Engineering (PIME) is starting to be employed recently, to build up an interfacial layer on polymeric fibre surfaces; this interfacial layer services as a platform to anchor catalysts for the following metal Electroless Deposition (ELD). The designed interfacial layer significantly increases adhesion between polymeric substrates and coating metal layers, to improve the durability of e-textiles. This review highlights recent research into different molecular and architectural design strategies, and its potential application for wearable electronics. Further challenges and opportunities in this field are also discussed critically.

## 1. Introduction

In recent years, researchers have shown an increased interest in wearable electronics, which have a wide range of applications, such as photodynamic therapy, flexible batteries, heating fabrics and wearable displays [1,2,3,4,5,6,7,8,9,10,11,12,13,14]. The fabrication of conductive components into flexible electronics is a key problem in achieving large scale production of smart textiles. There are a large number of published studies that report on conductors integrated with flexible substrates such as carbon materials, conducting polymers and metal [4,9,15]. Carbon black is an intrinsic black product which causes negative effects to the aesthetics of textiles. Graphene has no scaling up products and people are still seeking efficient technology in the exploitation of single layer graphene. Conductive polymers are very expensive and have relatively lower conductivity. Among these materials, metal is the most popular conductive material because it is cheap, highly conductive and widely compatible. For the intergradation of metal materials into flexible substrates and fibres, improving adhesion force is the key for the durability of e-textile and wearable electronics.

**Figure 1:**
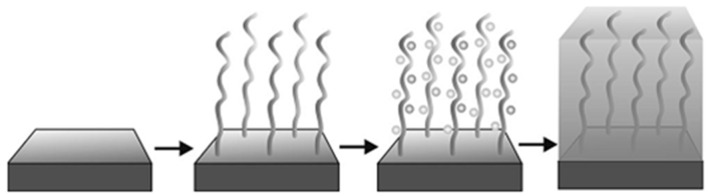
The conventional process for PIME catalytic ELD [4].

Several studies have attempted to coat metal nanoparticles onto soft substrates through techniques such as vapour phase deposition [16], chemical vapour deposition [17,18,19] and electro-deposition [20,21,22]. However, the fabrication via such methods does not have chemical bonds or any other tethering force between the metallic layer and the original substrates. The poor adhesion between the coating layer and the substrates limits the widespread application of wearable devices. Electroless deposition (ELD) is a low cost and simple method [23,24] for fabricating metal films (Cu, Ni, Ag, Au, etc.) on soft and stretchable substrates such as rubber, plastic, leaves and fabrics [9,25,26]. The polymer interface plays an important role during the whole ELD process because the surface modification significantly increases the adhesion between metal nanoparticles and flexible substrates [27]. The conventional process for Polymer Interface Molecular Engineering (PIME) catalytic ELD is illustrated in Figure 1. The first step is to modify raw substrates by grafting a polymer onto them; the second step involves the immobilisation of captured catalysts; the third step entails electroless deposition, whereby metal nanoparticles grow on the catalysed area [4,9].

## 2. Polymer Interface and Catalytic Moiety

**Figure 2:**
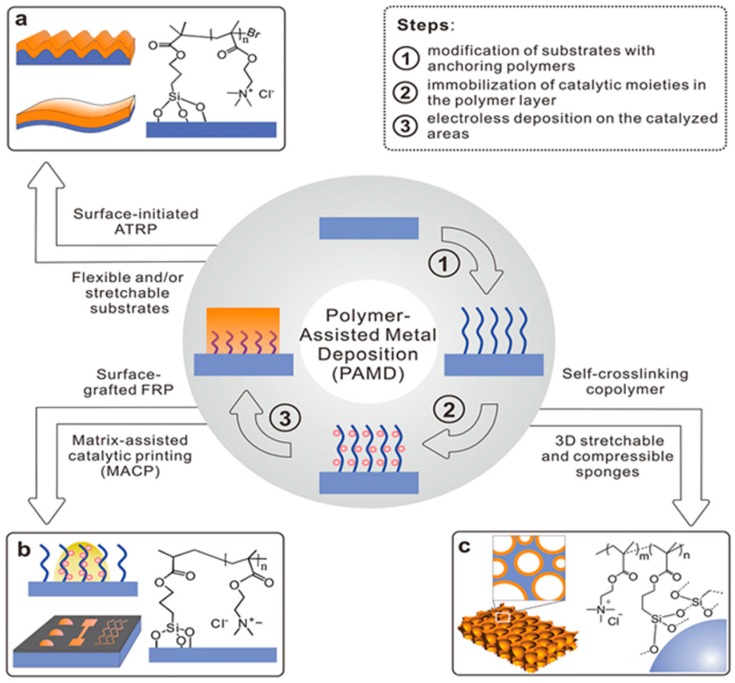
An illustration of polymerisation methods of the PIME catalytic ELD: (**a**) SI ATRP is applied on a flexible substrate; (**b**) In situ, FRP is conducted in situ via various printing techniques; (**c**) self-crosslinking copolymerisation is used on a three-dimensional (3D) electronic structure [9].

Recent decades have witnessed the development of various techniques for grafting polymers onto flexible substrates, such as self-crosslinking copolymerisation, free radical polymerisation (FRP), and surface-initiated atom transfer radical polymerisation (SI ATRP) [28,29], as shown in Figure 2. Additionally, there have been several academic studies on ELD relating to radiation-induced [30], plasma-induced [31] and UV-triggered polymerisation [32]. SI ATRP is a popular approach as it produces a high-density polymer layer; however, it also has some disadvantages, such as low productivity and a nitrogen atmosphere. FRP is conducted in an air environment and the thickness of the polymer interfacial layer ranges from tens to hundreds of nanometres [9]. Nevertheless, FRP requires pre-treatment (vinyl-terminating silane modification) before the polymerisation of poly[2-(methacryloyloxy)-ethyl]-trimethylammonium chloride (PMETAC), and can only produce conductive cotton yarns/fabrics without the plasma treatment.

Polyphenols, such as dopamine and tannic acid, are at the cutting edge of the PIME catalytic ELD field. Dopamine is a type of biomaterial found in mussels and can, through self-polymerisation in an aqueous solution at pH 8.5, form an adhesive film on a wide range of inorganic and organic materials, including fabrics, polymers and ceramics. More importantly, the robust film can continuously conduct various secondary treatments, including ELD at room temperature, without any specific equipment [33]. Tannic acid (TA) is usually extracted from plants such as gallnuts. Due to the numerous phenol groups in its structure, TA has excellent metal ions-chelating ability and can bind other materials via hydrogen bonds [34].

Modern polymer matrices (cationic, anionic, nonionic and polyphenols) can be used in PIME catalytic ELD for flexible electronic industries. Poly[2-(meth acryloy loxy)-ethyl]trimethylammonium chloride (PMETAC) [35,36,37,38,39] and poly(acrylic acid) (PAA) [40,41,42] are the two most common polymer interfaces, as both have the technical feasibility to synthesise and select marked catalytic moieties. Polyphenols are state-of-the-art polymer interfaces and have unexceptional properties for coating metal nanoparticles onto flexible and/or stretchable substrates.

### 2.1. Cationic Polymer Interface and Catalytic Moiety

**Figure 3:**
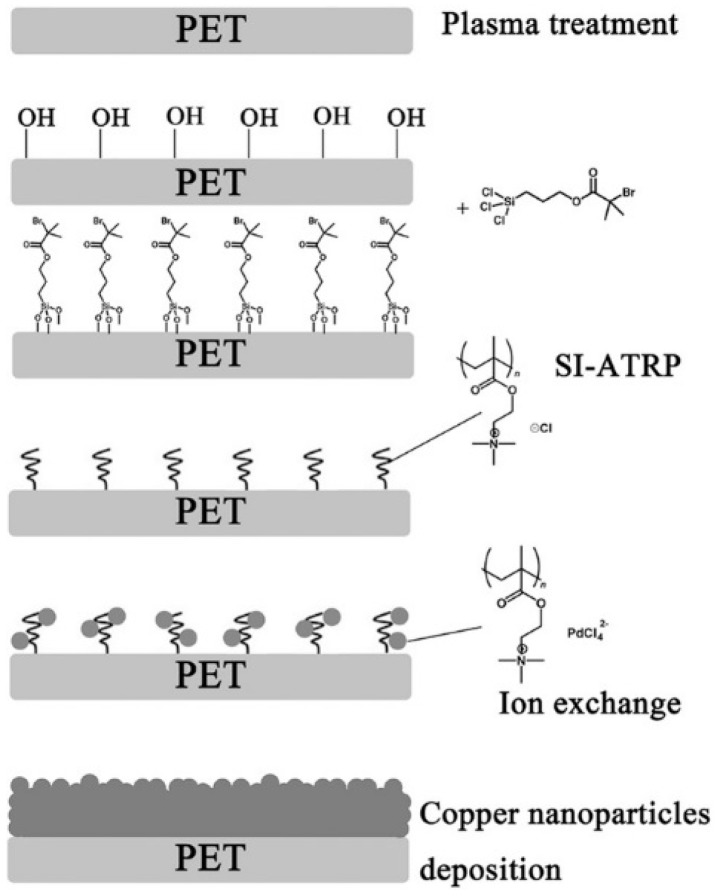
An illustration of the process of PMETAC-assisted copper ELD on PET substrates [43].

The cationic polymer interface is defined as a positively charged polyelectrolyte, which has a powerful affinity to the anionic catalytic moieties. PMETAC is a cationic polymer interface that is widely used to transfer ion species such as [PdCl_4_]^2−^. Liu et al. [43] reported that surface plasma treated poly(ethylene terephthalate) (PET) yarns/fabrics were immobilised with an ATRP initiator, and PMETAC was then employed to modify PET yarns/fabrics, after which catalytic moieties [PdCl_4_]^2−^ were loaded onto the fibre surface with the help of grafted PMETAC. The quaternary ammonium groups of PMETAC have a particular ability to anchor [PdCl_4_]^2−^ species, and catalytic areas were formed by ion exchange. The thin copper film was finally grown on the catalytic surface by immersing modified PET substrates into a copper ELD bath [43], as shown in Figure 3. The thickness of the copper film is determined by the amount of the catalyst, which depends on the concentration of [PdCl_4_]^2−^ species in the aqueous solution. The copper/PET hierarchical structures of the polymer bridges present excellent selectivity because deposition only takes place in the zone covered by the catalyst.

### 2.2. Anionic Polymer Matrix and Catalytic Moiety

**Figure 4:**
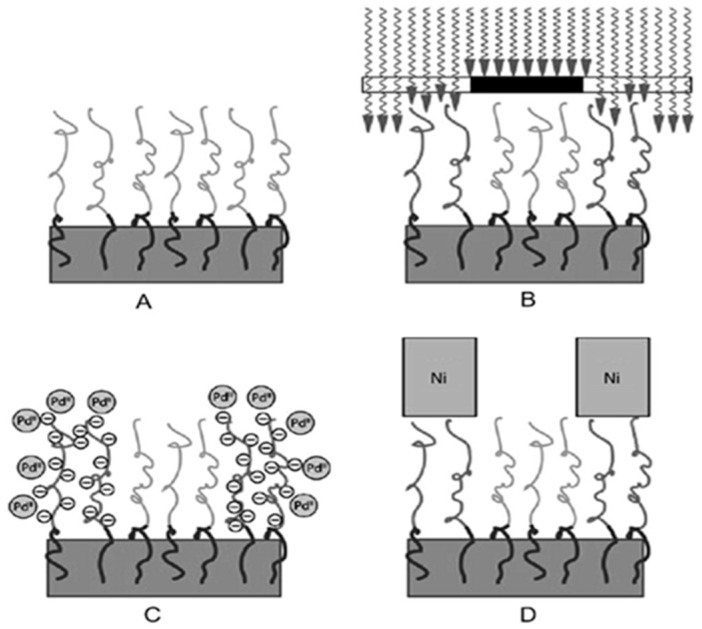
The process of ELD with diblock polymer PS-b-PtBA: (**A**) the copolymer was grafted onto the PS substrate; (**B**) PtBA blocks were transferred into PAA blocks under UV-radiation; (**C**) the catalytic ions were anchored by PAA brushes; (**D**) metal thin films formed in the catalytic areas [42].

The anionic polymer matrix is defined as a negatively charged polyelectrolyte which can immobilise the cationic moieties in the aqueous solution. The two most common anionic polymer models are poly(acrylic acid) (PAA) [40,41,42] and poly(meth acryloyl ethyl phosphate) (PMEP). PAA brushes negatively charged at an appropriate pH can tightly bind metal cations, such as Ni^2+^, Cu^2+^ and [Pd(NH_3_)_4_]^2+^ [4]. According to Kobersterin et al. [42], after a PAA interface catalytic ELD had been introduced, a block copolymer polystyrene-b-poly(tertbutyl acrylate) (PS-b-PtBA) was applied. In the first step, the polystyrene blocks penetrated a polystyrene substrate and the PtBA blocks were exposed to ultraviolet (UV) radiation, after which a photoacid generator hydrolysed them to yield PAA blocks. In the next step, PAA brushes captured catalytic ions ([Pd(NH_3_)_4_]^2+^) and a thin nickel layer was coated onto [Pd(NH_3_)_4_]^2+^ areas in a nickel ELD bath, as illustrated in Figure 4. In principle, this approach can be applied to all kinds of substrates, provided the original substrate and the first polymer block are the same.

**Figure 5:**
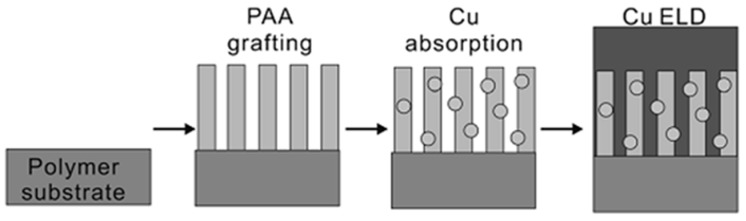
The process of ligand-induced electroless plating (LIEP) [40].

More recently, ligand-induced electroless plating (LIEP) was introduced for forming metal structures on polymer substrates [40,44,45]. To be precise, PAA was grown on polymer substrates via a GraftFast method, in which vinylic monomers were initiated by radical polymerisation in an aqueous solution (radicals were created by the reduction reaction of aryl- diazonium salts with iron powder) [46]. The PAA-modified substrate was immersed in a bath of alkaline solution containing Cu^2+^ ions and ammonia. Carboxylate groups of PAA then absorbed Cu^2+^ ions, and these absorbed ions were subsequently reduced by NaBH_4_. Finally, a thin copper film was fabricated on the polymer substrate, as shown in Figure 5. The LIEP system does not require an expensive palladium catalyst and can be used in many polymer substrates, such as polystyrene (PS), acrylonitrile-butadiene-styrene copolymer (ABS), polybutadiene (PB), poly(vinylidene fluoride) (PVDF), polyethylene terephthalate (PET) and polyacrylonitrile (PAN).

**Figure 6:**
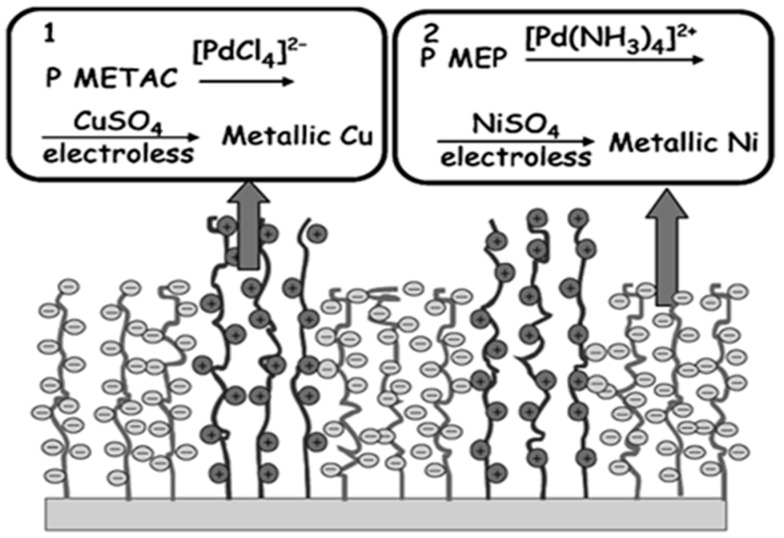
An explanation of mixed-polymer-matrix catalytic ELD. Positively and negatively charged polymers build highly selective copper and nickel layers [47].

PMEP, reported by Zheng, is another approach of anionic polymer matrix for ELD; the two oppositely charged polymer brushes have a strong affinity to various metal ions for site-selective ELD [47]. PMETAC and PMEP were synthesised by micro-contact printing and surface-initiated atom transfer radical polymerisation, respectively. [PdCl_4_]^2−^ species were captured via the quaternary ammonium groups of the PMETAC layer, which was positively charged, while [Pd(NH_3_)_4_]^2+^ ions were immobilised by the phosphate groups of PMEP chains, which were negatively charged. Two types of metal nanoparticles, Cu and Ni, were then deposited onto flexible substrates by immersing them into the ELD solution of copper and nickel, respectively, as shown in Figure 6. This method uses binary polymer brushes, acting as two seeding templates, to fabricate bimetallic layers on substrates.

### 2.3. Nonionic Polymer Interface and Catalytic Moiety

Nonionic polymers are another powerful polyelectrolyte with a strong affinity for related metal ionic catalysts of PIME catalytic ELD, including polyacrylonitrile (PAN), polyacrylamide (PAM), poly(vinyl pyrrolidone) (PVP), poly(2-vinyl pyridine) (P2VP) and poly(4-vinyl pyridine) (P4VP). There are two distinct properties of nonionic polymers: (1) In the presence of free nitrogen electron pairs, catalysts can be anchored onto the binding sites via ion exchange; (2) many polymers such as polyethylene (PE), polypropylene (PP), polyimide (PI), poly(tetrafluoroethylene) (PTFE), fluorinated polyimide (FPI) and poly-(tetrafluoroethylene-*co*-hexafluoropropylene) (FEP) can be used to modify substrates by various polymerisation techniques (radiation-, UV-, or plasma-induced polymerisation and SI ATRP). An advantage of this approach is that the polymerisation or copolymerisation of these nonionic polymers has the possibility of second processing, high productivity and large-scale production. More importantly, only simple devices are required to conduct the experiment.

**Figure 7:**
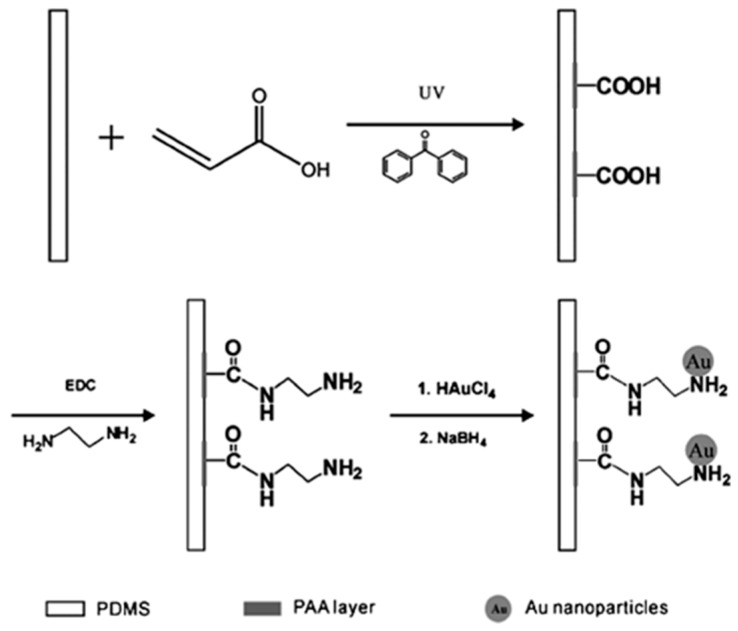
The process of ELD of a folded golden film via the treatment of post-modification of a surface-grafted PAA layer on a PDMS substrate [52].

Kang et al. [48,49,50] mentioned that N-containing nonionic polymer brushes could be polymerised on flexible substrates through post-modification of surface-grafted polymeric polyelectrolytes. Moreover, Yu et al. [51] found a strong adhesion after testing the metal layers that were plated onto these post-modified surface-grafted polymer brushes. Chen et al. [52] fabricated post-modified PAA into amine-terminating layers, which were then used to synthesise a gold film by ELD, as illustrated in Figure 7. UV-induced polymerisation was applied on a poly(dimethylsiloxane) (PDMS) substrate to trigger the synthesis of the PAA layer; ethylenediamine then modified the patterned PAA interface, which had carboxylic acid groups, to create new polymers with amine-terminating groups. These polymers were finally used to immobilise the gold nanoparticles. The results showed that a superior adhesion between the gold thin film and the PDMS surface was produced.

### 2.4. Polyphenols Interface and Catalytic Moiety

**Figure 8:**
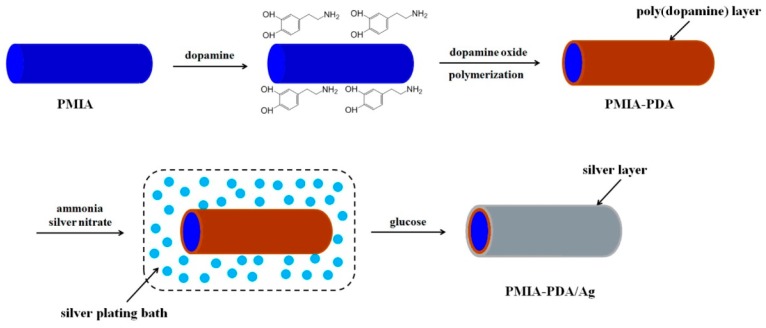
Schematic illustration of the production of PMIA-PDA-Ag fibres by PDA-assisted electroless silver deposition [53].

Polyphenols are a totally new polyelectrolyte, which have the strong ability to chelate related catalysts. The two most popular polyphenols applied in ELD are dopamine and tannic acid, whose unparalleled properties and attributes offer them a bright future in PIME catalytic ELD. Wang et al. [53] reported research of dopamine-assisted ELD, as shown in Figure 8. Polymetapheny-lene isophthamide (PMIA) fibres were coated with a layer of polydopamine (PDA) by immersing PMIA into an alkaline dopamine solution. This adhesive film then immobilised silver ions by chemical bonding. Glucose was subsequently added to the silver electroless plating bath to reduce silver ions into silver nanoparticles, and finally formed a silver layer on the PMIA surface. PDA-based PIME catalytic ELD is a simple, efficient and eco-friendly method.

**Figure 9:**
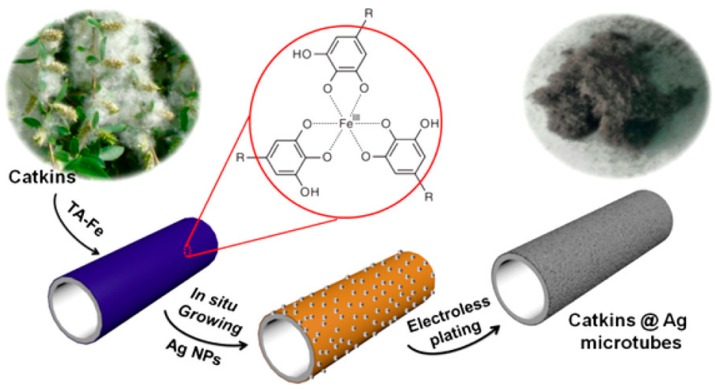
The process of the fabrication of Catkins-Ag microtubes by TA-assisted ELD [34].

Li et al. [34] presented research related to Tannic Acid (TA), as illustrated in Figure 9. According to their report, TA formed intermolecular hydrogen bonds with catkin fibres, which have hydroxyl groups that are advantageous for capturing TA-Fe coordination complexes on the catkin surface. Silver nanoparticles were then formed in situ on the fibre surface, acting as nucleation sites for later electroless silver coating. The TA polymer interface is firmly immobilised by chemical and/or physical reactions during the ELD process. More importantly, the polymer chains which have specific ligands can anchor the silver catalytic ions and yield robust adhesion. This technique has some advantages over PDA, although the PDA interface can form on many flexible solid substrates at room temperature without any pre-treatments, dopamine is expensive and the self-assembly of PDA is time consuming.

## 3. Substrates and Applications of ELD in Wearable Electronics

PIME catalytic ELD has been introduced as a highly productive method for fabricating metal conductors in flexible and stretched electronics [9]. Crucial components of this technique include not only the polymer interfaces and catalytic moieties, but also the different kinds of substrates used (metals, glass, rubbers, cotton and some biomimetic materials) [10], which have a significant impact on the properties of ELD electronics.

### 3.1. ELD on Plastic Substrates

**Figure 10:**
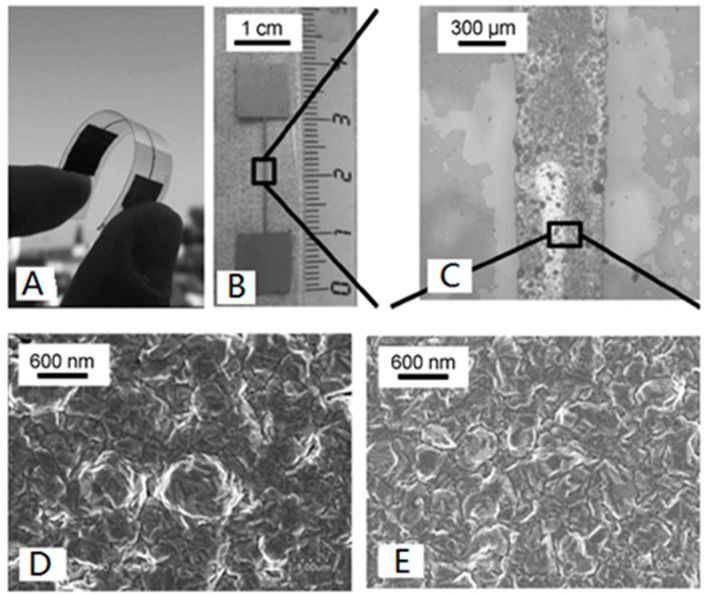
(**A**)/(**B**) are photographs of the PET sheet with the copper film; (**C**) is an optical image of the part in (**B**); (**D**)/(**E**) are Scanning Electron Microscopy (SEM) pictures of the copper structures on substrates of PET (**D**) and PVDF (**E**) [45].

PIME catalytic ELD is synthesised under simple conditions involving room temperature treatment and cheap equipment [23,24], which is ideal for plastic substrates. One report described how a thin copper electrode was formed through ELD, with the help of ligand-induced electroless plating [45]. PAA acted as a polymer platform grafted onto the surface of a poly (ethylene terephthalate) (PET) substrate to immobilise catalytic moieties and, later, the layer of copper deposited on catalytic areas. The formed copper electrode was robust and presented a high level of electrical conductivity. Another merit of this product is that the metal structures on the flexible PET substrate have desirable mechanical properties and are highly durable. As shown in Figure 10, the electrical conductive and mechanical properties of Cu structures on the substrates remained nearly the same after being bent 30 times.

### 3.2. ELD on Elastomer Substrates

**Figure 11:**
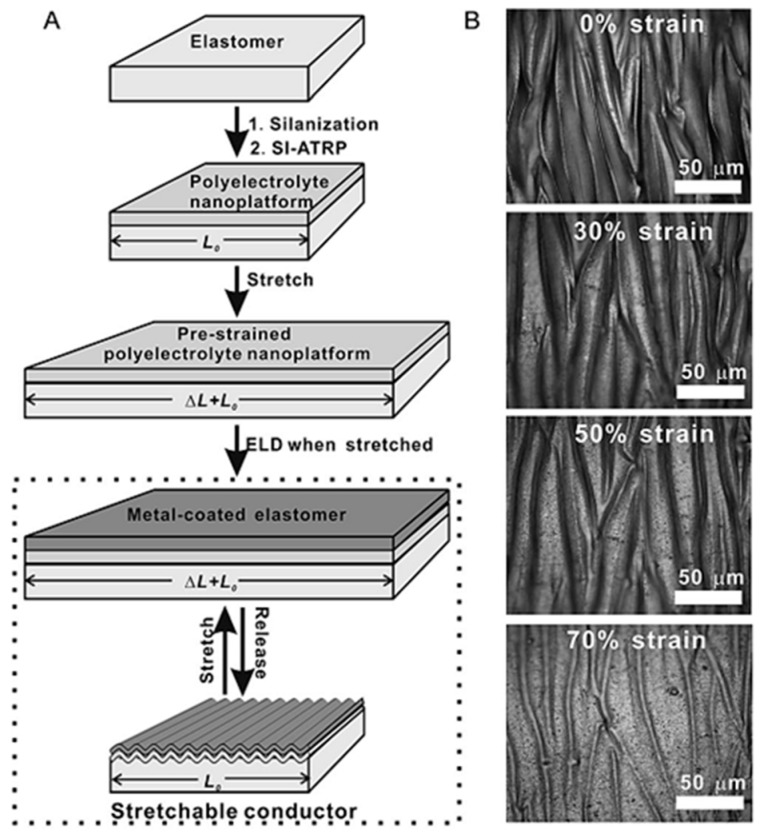
(**A**) An illustration of the synthesis process of a stretchable conductor on an elastomer substrate; (**B**) the optical microscopy images of copper layers of ELD on rubber at different tensile strains (from 0% to 70%) [37].

Elastomer materials are one of most popular substrates when applying ELD to produce smart textiles; however, their potential drawback is poor adhesion. Elastomer substrates can be stretched but the deposited metal layer cannot. Fortunately, Zheng et al. [37] tackled this issue and produced an outstanding stretchable conductor by ELD, to fabricate a copper thin film on a pre-strained elastomer substrate (Figure 11).

**Figure 12:**
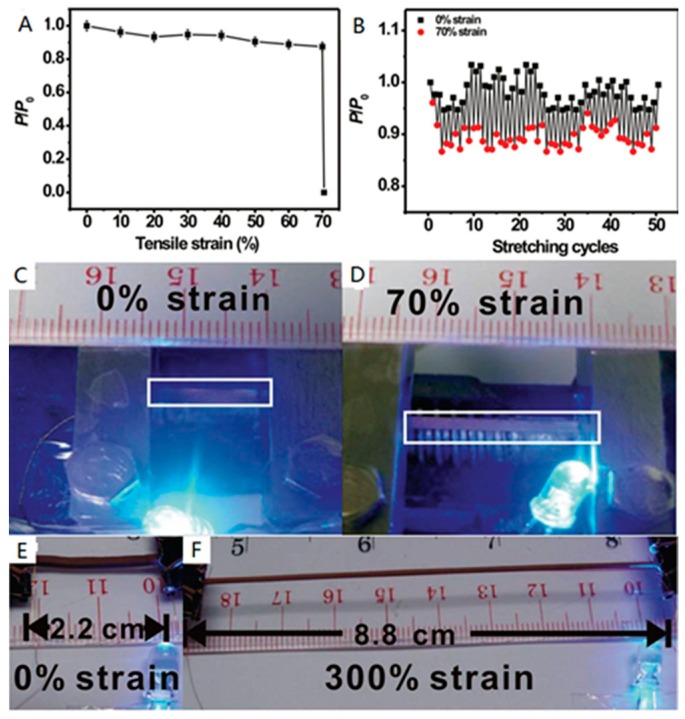
(**A**) The changes in conductivity of the high stretchable conductor at different tensile strains; (**B**) the changes of durability in conductivity at various stretching cycles when the tensile strains are 0% and 70%, respectively; (**C**)/(**D**) digital images of the high stretchable conductor linked with a light-emitting diode at 0% (**C**) and 70% (**D**) tensile strains; (**E**)/(**F**) digital images of the copper film with rubber substrate at 0% (**E**) and 300% (**F**) tensile strains [9,37].

PMETAC, as a polymer interface, was first grafted onto a rubber material, after which catalysts were immobilised, and this modified substrate was then stretched and immersed in a bath of copper ELD. Finally, the thin copper coating was grown on the surface of the pre-strain elastomer, and when the stress was removed, the rubber slice with copper layers could return to its initial shape. Additionally, when the as-made elastomer was stretched again (up to 300%), the metal layer was able to maintain good conductivity without any cracked coating [9,37]. As shown in Figure 12, the conductors are durable because of the robust adhesion between metal particles and the elastomer surface. This method thus solves the issue of low binding strength identified in prior reports, by producing a stretchable metal layer on the rubber substrate.

### 3.3. ELD on Cotton Substrates

**Figure 13:**
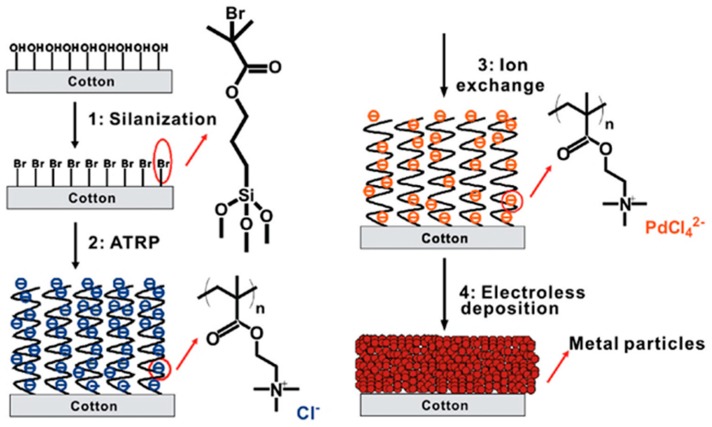
The processes of fabricating the metal particles on the cotton substrate through ELD [54].

**Figure 14:**
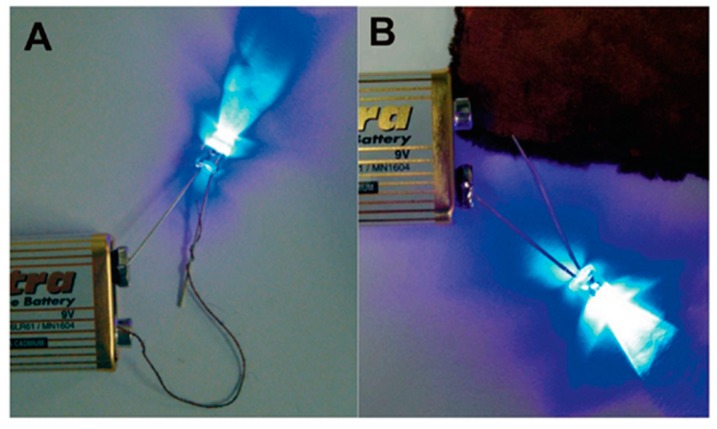
Digital images show an electrical wire (**A**) and conductive yarn (**B**) that are both linked with an LED [54].

Cotton, as one of the most universal natural fibres, has many advantages. For example, it is cheap and does not require complex processing systems. Because of these advantages, cotton has widespread application in our everyday life, including for garments, upholstery, socks and smart textiles [36]. Using natural fibres like cotton to produce flexible and comfortable electronics is more eco-friendly than artificial fibres. Zheng et al. reported a novel, straightforward and versatile technique to fabricate conductive metals on cotton substrates [9,36]. The whole process is illustrated in Figure 13. The bromine-terminating silane, which played the role of creating subsequently reacted sites for the polymer interface, first modified the surface of the cotton substrate, after which surface-initiated atom transfer radical polymerisation was used to graft the PMETAC matrix onto initiating points. These long polymer chains, which have a high density of binding sites, captured [PdCl_4_]^2−^ ions in an aqueous solution by exchanging ions. Cu or Ni particles were finally plated in catalytic regions [54]. A light-emitting diode (LED) was used to test the conductivity of the copper film, the results show that it has excellent electrically conductive property even when bent, stretched or washed (Figure 14).

### 3.4. ELD on Bio-Inspired Substrates

**Figure 15:**
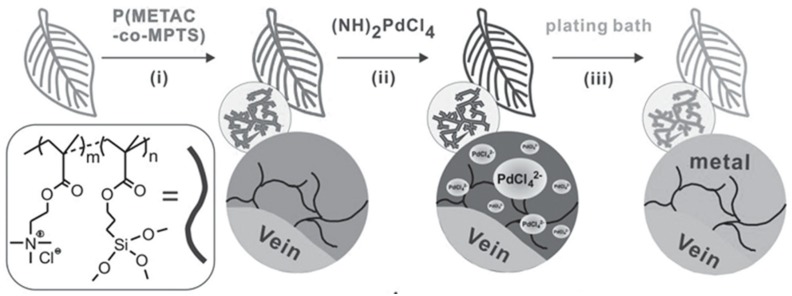
The process of ELD to produce vein-based transparent electrodes [10].

**Figure 16:**
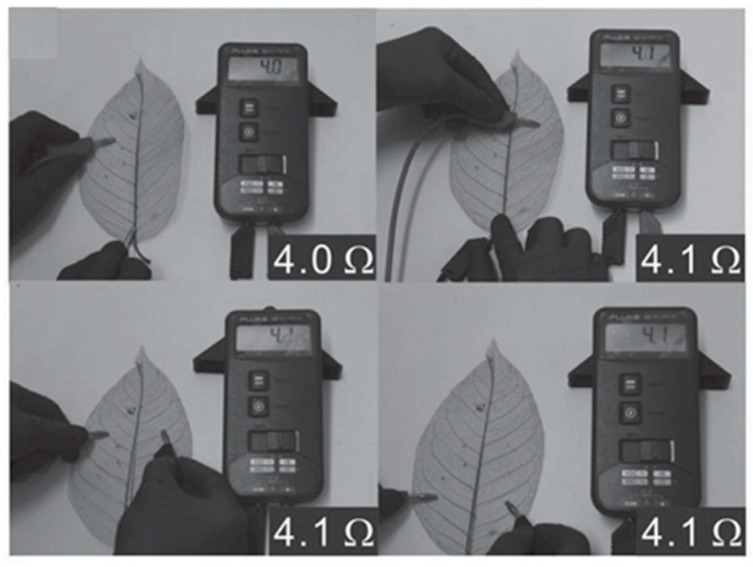
Random test about bulk resistances in different parts of the vein conductor [10].

**Figure 17:**
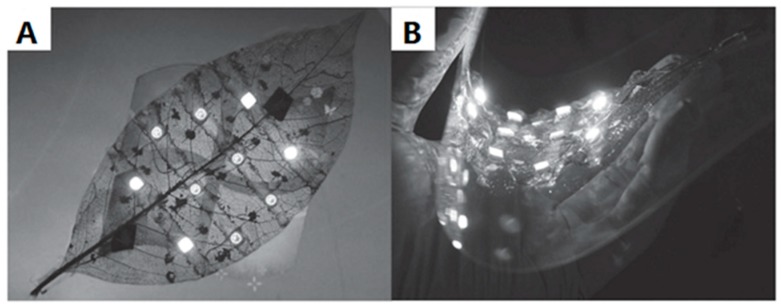
Glue was used to attach LEDs to the modified seam; it worked well when the wire stayed in various shapes: (**A**) flat vein-based transparent electrodes (**B**) bended vein conductors [10].

Nowadays, the ELD field tends to choose some bionic materials as an omnidirectional substrate, because natural fibres are green and cheap [10,25]. For instance, soft and stretchable plant leaves are suitable to act as substrates to be coated with a layer of metals by ELD. Recently, Zheng et al. [10] reported a method to fabricate vein-based transparent electrodes. A type of copolymer, P(METAC-*co*-MPTS), first modified the surfaces of natural veins; then these leaves, modified with polymer matrix, were immersed in a solution of (NH_4_)_2_PdCl_4_ for 2 h, after which [PdCl_4_]^2−^ ions were captured for subsequent depositing. Finally, copper particles were deposited on the surface of vein substrates with the help of the plating bath (Figure 15). The electrode that was produced has excellent electrically conductive attributes and is nearly transparent. As Figure 16 shows, measuring different parts of the vein conductor reveals that resistances of thread conductor bulks are about 4 Ω, which is superior to some trading products. Also, when different strains were applied to the vein-based conductor and integrated with the LED, it could still, as a powering electronic device, work in the circuit (Figure 17).

### 3.5. ELD on Three-Dimensional Substrates

**Figure 18:**
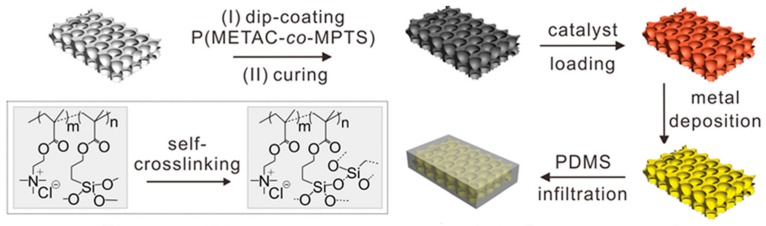
Processes of creating a three-dimensional metal conductor through polymer-matrix catalytic ELD [39].

**Figure 19:**
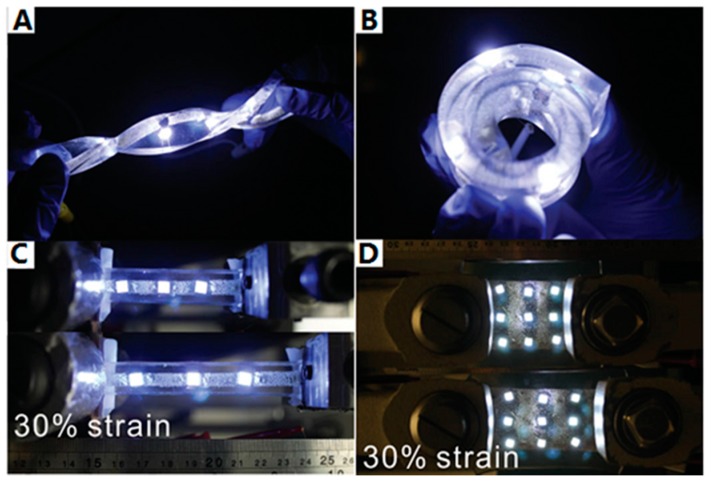
Digital images of conductive three-dimensional sponges with working LEDs on the surface at various strains and shapes: (**A**) twisted (**B**) bended (**C**) stretched at 30% strain (**D**) compressed at 30% strain [39].

**Figure 20:**
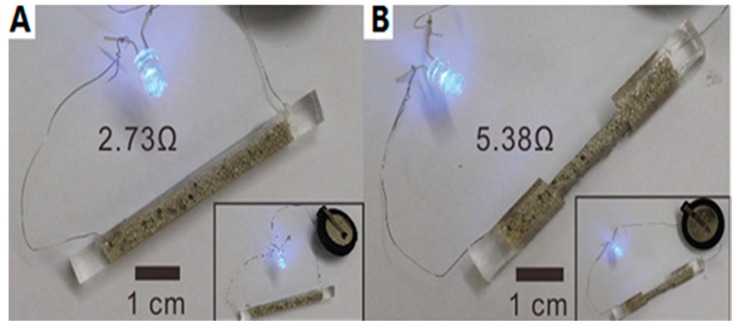
Illustration of the conductive three-dimensional sponges before (**A**) and after (**B**) cutting some parts away [39].

In previous reports, the PIME catalytic ELD only used two-dimensional conductors; however, Yu et al. [39] have introduced a new method to fabricate a flexible and stretchable three-dimensional wire on a porous polyurethane (PU) sponge. Unlike direct polymerisation on the substrate surface, the copolymer P(METAC-*co*-MPTS) was grafted by a dip coating and curing process. This approach can be roughly divided into three steps (Figure 18). (1) Bulk substrates were first dipped into an ethanol solution of P(METAC-*co*-MPTS) in the air-dried condition with bubbling of nitrogen gas; during the curing process, a connected layer formed by silane groups in the MPTS blocks built a robust adhesion between the polymer matrix and the PU sponges. (2) After immersing the surface-modified sponges in the catalyst solution, [PdCl_4_]^2−^ ions were immobilised by PMETAC blocks. (3) A thin layer of metal film (copper, copper/silver, gold) was generated on the surface of the PU sponges by ELD in the plating bath. The three-dimensional electrodes that were produced presented conductive, flexible and durable properties. The main advantage is that 3D structures can protect the conductive surface from damage more effectively than two-dimensional ones, which could be proved by a 3D-network conductive pathway [39]. Figure 19 and Figure 20 show that resistance did not change significantly after polishing with abrasive paper or cutting in half by a razor blade.

## 4. Surface Lithography by PIME Catalytic ELD

The most popular catalytic moieties for the PIME catalytic ELD technique are metal ions or nanoparticles [9]. The two most common methods for loading these catalytic moieties are immersing the substrates with polymer-modified surfaces into a metal-based salts solution, and printing the ink containing catalytic metal ions onto the modified surfaces. Although the first method is widely applied in many reports, printing techniques still play a fairly significant role in producing ELD wearable devices, because of their low cost and simplicity. Accordingly, it is important to identify advanced printing approaches through which cheap and high performance flexible ELD conductors can be fabricated. Nevertheless, due to the limitation of the viscosity problem of catalyst ink, inkjet printing has only been applied in ELD patterning techniques; this has a negative influence on producing variety and efficiency. In order to find new printing methods to coat metal layers, an effective way should be found to solve the problem of low ink viscosity.

**Figure 21:**
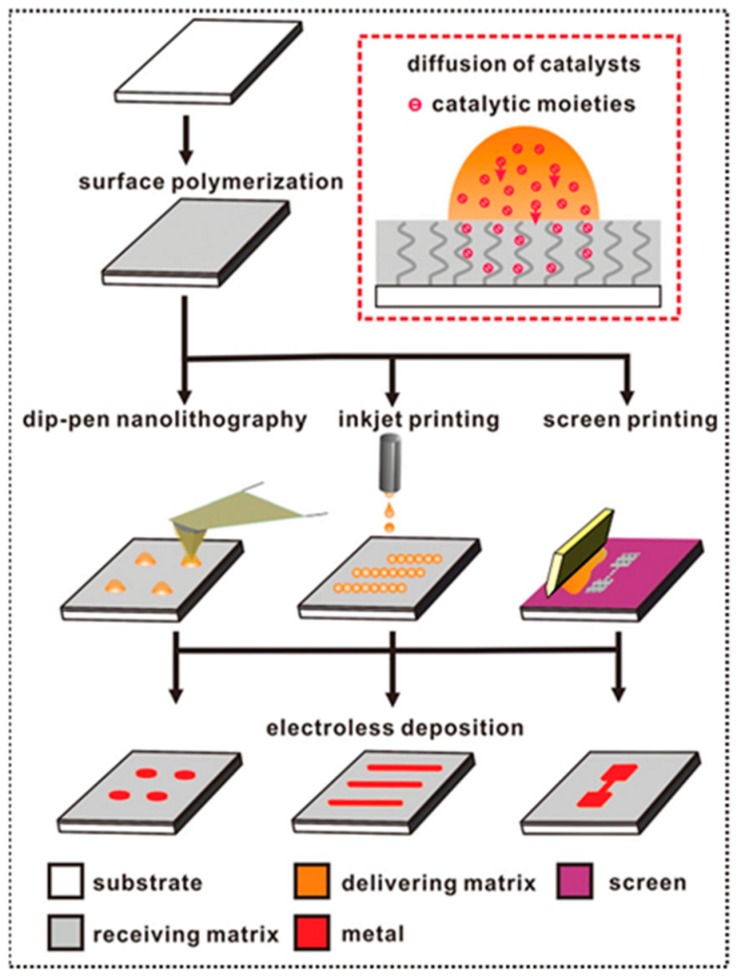
An illustration of ELD of metal films through dip-pen nanolithography, inkjet printing and screen printing [27].

Zheng et al. researched a novel approach to control the viscosity of the catalyst ink, by adding polyethylene glycol (PEG) as the delivering matrix [27]. In their report, the matrix-assisted catalytic printing (MACP) method was able to fabricate four metal conductors (copper, nickel, silver and gold) into different flexible substrates. The catalytic moieties were printed onto the substrate surface through three printing techniques—dip-pen nanolithography (DPN), inkjet printing and screen printing—rather than through immersing substrates directly in the catalyst solution or traditional lithography printing. As shown in Figure 21, PMETAC, which worked as a receiving platform, was grafted onto the substrate surface. The ink containing the catalyst solution and delivering matrix was then printed by three different printing methods. After ELD, the metal thin films formed on the printed areas. This method exhibited great selectivity and stability when transferring patterns from the ink to the metal layer.

The delivering matrix (PEG) played a vital role in these printing techniques [9,27]. The viscosity of the ink could be controlled effectively by the amount of PEG. For screen printing, the catalyst ink should be much more viscous than for dip-pen nanolithography (DPN) and inkjet printing. On the other hand, the water-absorbing PEG kept the ink’s moisture levels high to ensure that PMETAC had sufficient time to capture [PdCl_4_]^2−^ ions during the process of diffusion. Therefore, the ELD of metal films could be fabricated through various printing approaches. In addition, these flexible and stretchable conductors illustrated good mechanical properties and stable electrical properties. The minimum resistivity of the four conductors (copper) was just 4.9 µΩ·cm [27].

## 5. Conclusions

PIME catalytic ELD is a versatile and efficient way of fabricating metal nanoparticles or thin films onto substrates to produce flexible and stretchable electronics. Generally, this low-cost, convenient technique contains three steps to achieve metal conductors: Modifying substrates with polymer interfaces; capturing catalytic moieties on the polymer-grafted areas; and subsequently depositing the metal layer in an ELD bath. The substrates applied for ELD can be chosen from a wide range of materials, such as plastics, elastomers, cotton, PET, three-dimensional sponges and even natural leaves. Some previously reported substrates, polymer interfaces and polymerisation methods employed for PIME catalytic ELD, are summarised in Table 1.

The polymer interface is an important part of the whole process of fabrication because interpenetrating networks are formed between the polymer matrices layer and metal nanoparticles. More significantly, this interpenetration provides a powerful adhesion to overcome mechanical failures and instability, which minimises the cracking of the metal layer from the substrate surface when the conductors are stretched or folded. At the same time, the metal films fabricated from PIME catalytic ELD are usually high performance, with excellent conductive properties. In addition, as well as traditional lithographic approaches [66,67,68,69,70,71,72,73,74,75] (including photolithography, scanning probe lithography and contact printing), some advanced printing techniques have been applied in the ELD field. These effective printing methods, such as dip-pen nanolithography, inkjet printing and screen printing, have the potential to improve the throughput of patterned ELD flexible electronics.

However, research about PIME catalytic ELD remains at a preliminary stage [4,9]. Several drawbacks such as long reaction time and tedious synthesis steps of surface polymerization limit the industrial scaling up productions, and hinder practical applications. Setting up a novel polymeric system by surface molecular engineering, should be conducted in future. Moreover, main research works focus on conductive metal nanoparticles deposition. Other functional materials, such as semi-conductors, cannot be coated by PIME catalytic ELD. The deposition process is conducted in an aqueous solution; water pollution and sustainable development should be conceived as a critical factor. Additionally, in current reports, most ELD metal structures are only applied as simple conductive wires and electrodes. Developing high performance e-textiles, or conductive patterns using PIME, should start under multidisciplinary collaboration. More applications, such as robotic skins, point-of-care diagnostics, wearable displays and biological actuators, should be conducted in future.

## Figures and Tables

**Table 1:** Current results of PIME catalytic ELD.

Polymer Matrices	Polymerisation Method	Metal	Substrate
PMETAC [35,36,37,47]	SI-ATRP	Cu, Ni	Cotton, PET, PDMS, Au
PAA [40,42,44,45]	GraftFast, block copolymer	Cu, Ni	ABS, PET, PVDF, PAN, PB, PS
PVBVN [55]	Plasma grafting	Ag	PI
PMEP [47]	SI-ATRP	Cu, Ni	Au
PAA post-modified with ethylenediamine [52]	UV grafting	Au	PDMS
P2VP, P4VP [56,57,58,59,60,61,62,63,64]	UV, plasma, radiation grafting	Cu, Ni	PI, FPI, PTFE, silk, PVDF
PAM [56,65]	Radiation grafting	Cu	PP
PVP [65]	Radiation grafting	Cu	PE
PAN [51]	UV grafting	Cu	FEP
PHEA, PHMMAAm [50]	UV grafting	Cu	PTFE
PDDA [48,49]	Oxidative grafting	Cu	PTFE, glass
Dopamine [53]	Self-polymerisation	Ag	PIMA
Tannic Acid [34]	dip-coating	Ag	Catkin

PDDA = Poly(diallyldimethylammonium chloride), PVBVN = Poly-(1,1′-bis(4-vinylbenzyl)-4,4′-bipyridinium dinitrate), PHEA = Poly-2-hydroxyethyl acrylate, PHMMAAm = Poly-N-(hydroxymethyl) methacrylamide.
